# Supplementary material for: Modeling glioblastoma heterogeneity as a dynamic network of cell states
Source: Mol Syst Biol. 2021 Sep 16;17(9):e10105. doi: 10.15252/msb.202010105 (PMC8444284; doi:10.15252/msb.202010105)
Supplement: Supplementary file 6 — Source Data for Figure 5 [file MSB-17-e10105-s004.zip › Figure5A_sourcedata/GSEA_3071/nosigdb_stateV.GseaPreranked.1622795159954/RICHARDS_NOWAKOWSKI_SCIENCE_2017_MGE_IPC1_UPREG.html]

Details for gene set RICHARDS\_NOWAKOWSKI\_SCIENCE\_2017\_MGE\_IPC1\_UPREG[GSEA]

|  || Dataset | state43071 |
| Phenotype | NoPhenotypeAvailable |
| Upregulated in class | na\_pos |
| GeneSet | RICHARDS\_NOWAKOWSKI\_SCIENCE\_2017\_MGE\_IPC1\_UPREG |
| Enrichment Score (ES) | 0.5092856 |
| Normalized Enrichment Score (NES) | 3.490831 |
| Nominal p-value | 0.0 |
| FDR q-value | 0.0 |
| FWER p-Value | 0.0 |
Table: GSEA Results Summary

  

Fig 1: Enrichment plot: RICHARDS\_NOWAKOWSKI\_SCIENCE\_2017\_MGE\_IPC1\_UPREG      
 Profile of the Running ES Score & Positions of GeneSet Members on the Rank Ordered List

  

| PROBE | GENE SYMBOL | GENE\_TITLE | RANK IN GENE LIST | RANK METRIC SCORE | RUNNING ES | CORE ENRICHMENT || 1 | HIST1H1B |  |  | 0 | 2.500 | 0.0072 | Yes |
| 2 | HIST1H4C |  |  | 1 | 2.442 | 0.0143 | Yes |
| 3 | ATAD2 |  |  | 2 | 2.214 | 0.0207 | Yes |
| 4 | ESCO2 |  |  | 3 | 2.193 | 0.0270 | Yes |
| 5 | HMGB2 |  |  | 5 | 2.011 | 0.0326 | Yes |
| 6 | NUSAP1 |  |  | 6 | 2.001 | 0.0384 | Yes |
| 7 | HIST1H1C |  |  | 7 | 1.976 | 0.0441 | Yes |
| 8 | TOP2A |  |  | 14 | 1.842 | 0.0481 | Yes |
| 9 | HIST1H1E |  |  | 15 | 1.830 | 0.0534 | Yes |
| 10 | ASRGL1 |  |  | 16 | 1.819 | 0.0587 | Yes |
| 11 | HIST1H1D |  |  | 19 | 1.786 | 0.0634 | Yes |
| 12 | C21orf58 |  |  | 20 | 1.772 | 0.0685 | Yes |
| 13 | NCAPG |  |  | 21 | 1.760 | 0.0736 | Yes |
| 14 | RAD51AP1 |  |  | 22 | 1.757 | 0.0787 | Yes |
| 15 | CKB |  |  | 23 | 1.748 | 0.0837 | Yes |
| 16 | FAM111A |  |  | 24 | 1.738 | 0.0887 | Yes |
| 17 | H2AFX |  |  | 26 | 1.725 | 0.0935 | Yes |
| 18 | CENPF |  |  | 28 | 1.705 | 0.0982 | Yes |
| 19 | TYMS |  |  | 30 | 1.683 | 0.1029 | Yes |
| 20 | RRM2 |  |  | 34 | 1.660 | 0.1071 | Yes |
| 21 | CENPU |  |  | 36 | 1.645 | 0.1116 | Yes |
| 22 | ASCL1 |  |  | 43 | 1.604 | 0.1150 | Yes |
| 23 | MKI67 |  |  | 45 | 1.598 | 0.1194 | Yes |
| 24 | KIF15 |  |  | 52 | 1.568 | 0.1227 | Yes |
| 25 | FOXM1 |  |  | 53 | 1.550 | 0.1272 | Yes |
| 26 | PBK |  |  | 54 | 1.536 | 0.1316 | Yes |
| 27 | EZH2 |  |  | 55 | 1.534 | 0.1360 | Yes |
| 28 | MXD3 |  |  | 62 | 1.500 | 0.1391 | Yes |
| 29 | GTSE1 |  |  | 66 | 1.491 | 0.1428 | Yes |
| 30 | CDK1 |  |  | 69 | 1.488 | 0.1467 | Yes |
| 31 | SMC2 |  |  | 73 | 1.466 | 0.1503 | Yes |
| 32 | SPC25 |  |  | 76 | 1.442 | 0.1540 | Yes |
| 33 | UBE2T |  |  | 79 | 1.432 | 0.1577 | Yes |
| 34 | FANCD2 |  |  | 81 | 1.423 | 0.1616 | Yes |
| 35 | FBXO5 |  |  | 82 | 1.422 | 0.1657 | Yes |
| 36 | HES6 |  |  | 91 | 1.391 | 0.1681 | Yes |
| 37 | DEK |  |  | 94 | 1.384 | 0.1717 | Yes |
| 38 | CDCA2 |  |  | 96 | 1.382 | 0.1755 | Yes |
| 39 | KIFC1 |  |  | 113 | 1.338 | 0.1760 | Yes |
| 40 | TACC3 |  |  | 116 | 1.334 | 0.1795 | Yes |
| 41 | LMO1 |  |  | 122 | 1.326 | 0.1822 | Yes |
| 42 | CDCA5 |  |  | 123 | 1.326 | 0.1861 | Yes |
| 43 | SMC4 |  |  | 127 | 1.320 | 0.1893 | Yes |
| 44 | BARD1 |  |  | 132 | 1.310 | 0.1922 | Yes |
| 45 | TMSB15A |  |  | 135 | 1.306 | 0.1956 | Yes |
| 46 | RTKN2 |  |  | 143 | 1.285 | 0.1978 | Yes |
| 47 | GMNN |  |  | 146 | 1.280 | 0.2011 | Yes |
| 48 | SOX2 |  |  | 147 | 1.279 | 0.2048 | Yes |
| 49 | NCAPD2 |  |  | 157 | 1.252 | 0.2065 | Yes |
| 50 | CCNF |  |  | 158 | 1.250 | 0.2102 | Yes |
| 51 | MIS18BP1 |  |  | 159 | 1.247 | 0.2138 | Yes |
| 52 | POLQ |  |  | 160 | 1.245 | 0.2173 | Yes |
| 53 | TMPO |  |  | 161 | 1.243 | 0.2209 | Yes |
| 54 | CENPK |  |  | 162 | 1.241 | 0.2245 | Yes |
| 55 | CDKN3 |  |  | 163 | 1.239 | 0.2281 | Yes |
| 56 | LMNB1 |  |  | 165 | 1.238 | 0.2315 | Yes |
| 57 | KIF23 |  |  | 167 | 1.235 | 0.2348 | Yes |
| 58 | NCAPH |  |  | 168 | 1.235 | 0.2384 | Yes |
| 59 | CDCA3 |  |  | 169 | 1.230 | 0.2419 | Yes |
| 60 | DBF4 |  |  | 189 | 1.197 | 0.2415 | Yes |
| 61 | CDKN2D |  |  | 190 | 1.196 | 0.2449 | Yes |
| 62 | KIF4A |  |  | 191 | 1.193 | 0.2484 | Yes |
| 63 | NUF2 |  |  | 192 | 1.189 | 0.2518 | Yes |
| 64 | ZNF43 |  |  | 201 | 1.169 | 0.2535 | Yes |
| 65 | USP1 |  |  | 202 | 1.168 | 0.2569 | Yes |
| 66 | ZWINT |  |  | 204 | 1.166 | 0.2600 | Yes |
| 67 | ASPM |  |  | 207 | 1.163 | 0.2630 | Yes |
| 68 | PRC1 |  |  | 211 | 1.161 | 0.2657 | Yes |
| 69 | ARL6IP1 |  |  | 212 | 1.158 | 0.2690 | Yes |
| 70 | KIF22 |  |  | 213 | 1.157 | 0.2724 | Yes |
| 71 | UBE2C |  |  | 214 | 1.154 | 0.2757 | Yes |
| 72 | CEP97 |  |  | 221 | 1.146 | 0.2778 | Yes |
| 73 | CDKN2C |  |  | 223 | 1.143 | 0.2809 | Yes |
| 74 | NCAPG2 |  |  | 225 | 1.141 | 0.2840 | Yes |
| 75 | BTG3 |  |  | 226 | 1.141 | 0.2873 | Yes |
| 76 | NDC80 |  |  | 240 | 1.129 | 0.2878 | Yes |
| 77 | TPX2 |  |  | 248 | 1.122 | 0.2896 | Yes |
| 78 | RAD21 |  |  | 256 | 1.109 | 0.2914 | Yes |
| 79 | MYEF2 |  |  | 271 | 1.090 | 0.2916 | Yes |
| 80 | KIF14 |  |  | 272 | 1.090 | 0.2948 | Yes |
| 81 | HAUS8 |  |  | 273 | 1.089 | 0.2979 | Yes |
| 82 | BRCA2 |  |  | 274 | 1.089 | 0.3010 | Yes |
| 83 | CHD7 |  |  | 275 | 1.088 | 0.3042 | Yes |
| 84 | BUB1 |  |  | 277 | 1.084 | 0.3071 | Yes |
| 85 | ZBTB20 |  |  | 281 | 1.080 | 0.3096 | Yes |
| 86 | CKAP2L |  |  | 285 | 1.078 | 0.3121 | Yes |
| 87 | CDK5RAP2 |  |  | 292 | 1.076 | 0.3140 | Yes |
| 88 | ECT2 |  |  | 295 | 1.071 | 0.3166 | Yes |
| 89 | AURKB |  |  | 297 | 1.068 | 0.3195 | Yes |
| 90 | SUGP2 |  |  | 302 | 1.065 | 0.3218 | Yes |
| 91 | ARHGAP11A |  |  | 303 | 1.064 | 0.3248 | Yes |
| 92 | CCNA2 |  |  | 309 | 1.057 | 0.3268 | Yes |
| 93 | CKAP2 |  |  | 312 | 1.053 | 0.3295 | Yes |
| 94 | PRR11 |  |  | 317 | 1.048 | 0.3317 | Yes |
| 95 | WEE1 |  |  | 322 | 1.044 | 0.3339 | Yes |
| 96 | RHNO1 |  |  | 326 | 1.040 | 0.3362 | Yes |
| 97 | SORT1 |  |  | 335 | 1.030 | 0.3376 | Yes |
| 98 | MPPED2 |  |  | 338 | 1.025 | 0.3401 | Yes |
| 99 | JAM2 |  |  | 351 | 1.015 | 0.3405 | Yes |
| 100 | KIF11 |  |  | 369 | 0.982 | 0.3399 | Yes |
| 101 | MCAM |  |  | 370 | 0.980 | 0.3427 | Yes |
| 102 | RAB3IP |  |  | 371 | 0.980 | 0.3455 | Yes |
| 103 | APOLD1 |  |  | 372 | 0.980 | 0.3483 | Yes |
| 104 | MYBL1 |  |  | 375 | 0.977 | 0.3508 | Yes |
| 105 | PHF19 |  |  | 378 | 0.971 | 0.3531 | Yes |
| 106 | LRRC58 |  |  | 380 | 0.969 | 0.3557 | Yes |
| 107 | FRMD4B |  |  | 381 | 0.968 | 0.3585 | Yes |
| 108 | TTK |  |  | 383 | 0.966 | 0.3611 | Yes |
| 109 | PCM1 |  |  | 386 | 0.964 | 0.3635 | Yes |
| 110 | DEPDC1B |  |  | 387 | 0.963 | 0.3663 | Yes |
| 111 | UHRF1 |  |  | 390 | 0.958 | 0.3686 | Yes |
| 112 | CIT |  |  | 391 | 0.958 | 0.3714 | Yes |
| 113 | HJURP |  |  | 406 | 0.945 | 0.3712 | Yes |
| 114 | SPC24 |  |  | 414 | 0.938 | 0.3725 | Yes |
| 115 | DLGAP5 |  |  | 418 | 0.936 | 0.3745 | Yes |
| 116 | MAD2L1 |  |  | 421 | 0.932 | 0.3768 | Yes |
| 117 | SAP30 |  |  | 424 | 0.930 | 0.3791 | Yes |
| 118 | CEP192 |  |  | 429 | 0.924 | 0.3809 | Yes |
| 119 | KPNA2 |  |  | 435 | 0.919 | 0.3825 | Yes |
| 120 | CENPE |  |  | 437 | 0.918 | 0.3850 | Yes |
| 121 | LBR |  |  | 438 | 0.916 | 0.3876 | Yes |
| 122 | INCENP |  |  | 440 | 0.916 | 0.3901 | Yes |
| 123 | RACGAP1 |  |  | 443 | 0.912 | 0.3923 | Yes |
| 124 | VRK1 |  |  | 445 | 0.911 | 0.3947 | Yes |
| 125 | FAM83D |  |  | 447 | 0.909 | 0.3971 | Yes |
| 126 | ORC6 |  |  | 449 | 0.908 | 0.3995 | Yes |
| 127 | STIL |  |  | 455 | 0.903 | 0.4011 | Yes |
| 128 | KNSTRN |  |  | 457 | 0.902 | 0.4035 | Yes |
| 129 | SHCBP1 |  |  | 465 | 0.900 | 0.4047 | Yes |
| 130 | HMGN2 |  |  | 466 | 0.900 | 0.4073 | Yes |
| 131 | MGME1 |  |  | 469 | 0.892 | 0.4094 | Yes |
| 132 | DNA2 |  |  | 470 | 0.890 | 0.4120 | Yes |
| 133 | CENPJ |  |  | 479 | 0.885 | 0.4129 | Yes |
| 134 | MND1 |  |  | 480 | 0.885 | 0.4154 | Yes |
| 135 | SPAG5 |  |  | 481 | 0.884 | 0.4180 | Yes |
| 136 | MPHOSPH9 |  |  | 489 | 0.877 | 0.4191 | Yes |
| 137 | UBE2S |  |  | 514 | 0.854 | 0.4166 | Yes |
| 138 | TTF2 |  |  | 517 | 0.853 | 0.4186 | Yes |
| 139 | NLGN4X |  |  | 523 | 0.851 | 0.4200 | Yes |
| 140 | PLK1 |  |  | 526 | 0.850 | 0.4221 | Yes |
| 141 | LMNB2 |  |  | 534 | 0.846 | 0.4231 | Yes |
| 142 | BUB1B |  |  | 535 | 0.846 | 0.4255 | Yes |
| 143 | BIRC5 |  |  | 541 | 0.842 | 0.4269 | Yes |
| 144 | SALL1 |  |  | 545 | 0.836 | 0.4287 | Yes |
| 145 | CEP152 |  |  | 547 | 0.834 | 0.4309 | Yes |
| 146 | CDC25B |  |  | 566 | 0.820 | 0.4295 | Yes |
| 147 | MID1 |  |  | 574 | 0.815 | 0.4304 | Yes |
| 148 | CCDC77 |  |  | 581 | 0.811 | 0.4315 | Yes |
| 149 | HMGB1 |  |  | 583 | 0.810 | 0.4337 | Yes |
| 150 | DIAPH3 |  |  | 585 | 0.808 | 0.4358 | Yes |
| 151 | LCORL |  |  | 594 | 0.804 | 0.4365 | Yes |
| 152 | TRA2B |  |  | 596 | 0.802 | 0.4386 | Yes |
| 153 | CDK6 |  |  | 605 | 0.798 | 0.4392 | Yes |
| 154 | RFWD3 |  |  | 616 | 0.790 | 0.4394 | Yes |
| 155 | GEN1 |  |  | 619 | 0.789 | 0.4413 | Yes |
| 156 | HMMR |  |  | 622 | 0.788 | 0.4432 | Yes |
| 157 | CENPA |  |  | 627 | 0.786 | 0.4446 | Yes |
| 158 | PIF1 |  |  | 632 | 0.782 | 0.4460 | Yes |
| 159 | CENPL |  |  | 640 | 0.778 | 0.4468 | Yes |
| 160 | SRGAP2B |  |  | 648 | 0.774 | 0.4476 | Yes |
| 161 | PBX3 |  |  | 657 | 0.771 | 0.4482 | Yes |
| 162 | GKAP1 |  |  | 663 | 0.768 | 0.4494 | Yes |
| 163 | NUP62 |  |  | 664 | 0.767 | 0.4516 | Yes |
| 164 | COX17 |  |  | 666 | 0.766 | 0.4536 | Yes |
| 165 | SKA3 |  |  | 668 | 0.765 | 0.4556 | Yes |
| 166 | C18orf54 |  |  | 669 | 0.765 | 0.4578 | Yes |
| 167 | NDE1 |  |  | 672 | 0.762 | 0.4596 | Yes |
| 168 | KIF18A |  |  | 679 | 0.759 | 0.4605 | Yes |
| 169 | CDC25C |  |  | 684 | 0.755 | 0.4619 | Yes |
| 170 | GAS2L3 |  |  | 685 | 0.755 | 0.4641 | Yes |
| 171 | CENPO |  |  | 686 | 0.755 | 0.4662 | Yes |
| 172 | KIF18B |  |  | 698 | 0.747 | 0.4661 | Yes |
| 173 | GPX4 |  |  | 701 | 0.746 | 0.4678 | Yes |
| 174 | RHOBTB3 |  |  | 705 | 0.744 | 0.4694 | Yes |
| 175 | ANP32A |  |  | 707 | 0.743 | 0.4713 | Yes |
| 176 | SMC3 |  |  | 708 | 0.743 | 0.4735 | Yes |
| 177 | PLK4 |  |  | 720 | 0.739 | 0.4733 | Yes |
| 178 | ANP32E |  |  | 722 | 0.739 | 0.4752 | Yes |
| 179 | HNRNPUL1 |  |  | 725 | 0.735 | 0.4770 | Yes |
| 180 | TEX30 |  |  | 730 | 0.730 | 0.4782 | Yes |
| 181 | FAT1 |  |  | 740 | 0.727 | 0.4785 | Yes |
| 182 | CKAP5 |  |  | 752 | 0.723 | 0.4783 | Yes |
| 183 | AURKA |  |  | 761 | 0.718 | 0.4787 | Yes |
| 184 | DCP2 |  |  | 762 | 0.717 | 0.4808 | Yes |
| 185 | CCDC150 |  |  | 773 | 0.713 | 0.4808 | Yes |
| 186 | TUBG1 |  |  | 776 | 0.712 | 0.4824 | Yes |
| 187 | ACTL6A |  |  | 780 | 0.709 | 0.4838 | Yes |
| 188 | CBX5 |  |  | 783 | 0.708 | 0.4854 | Yes |
| 189 | PSRC1 |  |  | 787 | 0.705 | 0.4869 | Yes |
| 190 | CENPC |  |  | 797 | 0.702 | 0.4870 | Yes |
| 191 | DTYMK |  |  | 800 | 0.700 | 0.4886 | Yes |
| 192 | TBC1D31 |  |  | 801 | 0.700 | 0.4907 | Yes |
| 193 | RSRC1 |  |  | 818 | 0.693 | 0.4893 | Yes |
| 194 | RBBP7 |  |  | 829 | 0.687 | 0.4893 | Yes |
| 195 | TUBB |  |  | 836 | 0.685 | 0.4900 | Yes |
| 196 | TGIF1 |  |  | 840 | 0.683 | 0.4913 | Yes |
| 197 | H2AFZ |  |  | 851 | 0.677 | 0.4912 | Yes |
| 198 | RDX |  |  | 858 | 0.675 | 0.4919 | Yes |
| 199 | SKA1 |  |  | 868 | 0.672 | 0.4920 | Yes |
| 200 | NUP50 |  |  | 875 | 0.668 | 0.4927 | Yes |
| 201 | CHEK2 |  |  | 882 | 0.667 | 0.4934 | Yes |
| 202 | NEK2 |  |  | 901 | 0.659 | 0.4916 | Yes |
| 203 | MELK |  |  | 915 | 0.653 | 0.4907 | Yes |
| 204 | MZT1 |  |  | 917 | 0.652 | 0.4924 | Yes |
| 205 | NEIL3 |  |  | 919 | 0.651 | 0.4941 | Yes |
| 206 | JADE1 |  |  | 931 | 0.647 | 0.4937 | Yes |
| 207 | H2AFV |  |  | 933 | 0.647 | 0.4953 | Yes |
| 208 | KIF20B |  |  | 934 | 0.647 | 0.4972 | Yes |
| 209 | PTTG1 |  |  | 936 | 0.647 | 0.4989 | Yes |
| 210 | LIN54 |  |  | 938 | 0.646 | 0.5005 | Yes |
| 211 | H3F3A |  |  | 944 | 0.643 | 0.5014 | Yes |
| 212 | DCAF7 |  |  | 951 | 0.639 | 0.5020 | Yes |
| 213 | CENPN |  |  | 953 | 0.638 | 0.5036 | Yes |
| 214 | ZFAND6 |  |  | 958 | 0.638 | 0.5046 | Yes |
| 215 | CKS2 |  |  | 966 | 0.634 | 0.5050 | Yes |
| 216 | SEMA5A |  |  | 974 | 0.630 | 0.5053 | Yes |
| 217 | LRR1 |  |  | 991 | 0.623 | 0.5038 | Yes |
| 218 | ZNF680 |  |  | 1004 | 0.619 | 0.5031 | Yes |
| 219 | CNTRL |  |  | 1011 | 0.617 | 0.5037 | Yes |
| 220 | TUBA1B |  |  | 1012 | 0.617 | 0.5055 | Yes |
| 221 | TERF1 |  |  | 1017 | 0.616 | 0.5064 | Yes |
| 222 | MIS18A |  |  | 1023 | 0.614 | 0.5071 | Yes |
| 223 | MNS1 |  |  | 1034 | 0.613 | 0.5068 | Yes |
| 224 | CDCA8 |  |  | 1043 | 0.610 | 0.5069 | Yes |
| 225 | NMU |  |  | 1056 | 0.605 | 0.5062 | Yes |
| 226 | FANCM |  |  | 1059 | 0.604 | 0.5075 | Yes |
| 227 | CDC7 |  |  | 1060 | 0.604 | 0.5093 | Yes |
| 228 | FIGN |  |  | 1093 | 0.594 | 0.5044 | No |
| 229 | RBMX |  |  | 1095 | 0.594 | 0.5059 | No |
| 230 | BCHE |  |  | 1116 | 0.588 | 0.5034 | No |
| 231 | ING1 |  |  | 1122 | 0.587 | 0.5041 | No |
| 232 | KIF20A |  |  | 1133 | 0.583 | 0.5037 | No |
| 233 | SPATA5 |  |  | 1139 | 0.581 | 0.5044 | No |
| 234 | FAM122B |  |  | 1143 | 0.579 | 0.5054 | No |
| 235 | HMGB3 |  |  | 1154 | 0.577 | 0.5050 | No |
| 236 | NUCKS1 |  |  | 1178 | 0.570 | 0.5019 | No |
| 237 | DEPDC1 |  |  | 1179 | 0.569 | 0.5035 | No |
| 238 | ITGB3BP |  |  | 1180 | 0.568 | 0.5052 | No |
| 239 | ANP32B |  |  | 1187 | 0.566 | 0.5056 | No |
| 240 | HP1BP3 |  |  | 1198 | 0.562 | 0.5051 | No |
| 241 | PARPBP |  |  | 1210 | 0.560 | 0.5044 | No |
| 242 | GNG11 |  |  | 1219 | 0.556 | 0.5044 | No |
| 243 | MTFR2 |  |  | 1222 | 0.555 | 0.5056 | No |
| 244 | EXOSC8 |  |  | 1230 | 0.553 | 0.5057 | No |
| 245 | CTDSPL2 |  |  | 1256 | 0.544 | 0.5021 | No |
| 246 | CEP135 |  |  | 1264 | 0.541 | 0.5022 | No |
| 247 | SLC4A8 |  |  | 1266 | 0.540 | 0.5036 | No |
| 248 | TFDP2 |  |  | 1268 | 0.540 | 0.5049 | No |
| 249 | ARHGEF39 |  |  | 1285 | 0.536 | 0.5032 | No |
| 250 | SRSF10 |  |  | 1289 | 0.535 | 0.5041 | No |
| 251 | HMGN5 |  |  | 1292 | 0.534 | 0.5052 | No |
| 252 | CCDC167 |  |  | 1303 | 0.531 | 0.5047 | No |
| 253 | CCDC18 |  |  | 1305 | 0.530 | 0.5060 | No |
| 254 | KIF2C |  |  | 1309 | 0.530 | 0.5069 | No |
| 255 | NES |  |  | 1325 | 0.527 | 0.5053 | No |
| 256 | PIK3R3 |  |  | 1327 | 0.526 | 0.5067 | No |
| 257 | CENPI |  |  | 1351 | 0.519 | 0.5034 | No |
| 258 | METTL7A |  |  | 1361 | 0.517 | 0.5030 | No |
| 259 | NDUFAF3 |  |  | 1363 | 0.516 | 0.5043 | No |
| 260 | CNBP |  |  | 1370 | 0.514 | 0.5045 | No |
| 261 | CCNB2 |  |  | 1373 | 0.513 | 0.5056 | No |
| 262 | LIN9 |  |  | 1376 | 0.512 | 0.5067 | No |
| 263 | DZIP3 |  |  | 1384 | 0.510 | 0.5067 | No |
| 264 | NUP107 |  |  | 1388 | 0.509 | 0.5075 | No |
| 265 | SPDL1 |  |  | 1397 | 0.507 | 0.5073 | No |
| 266 | SAPCD2 |  |  | 1455 | 0.491 | 0.4970 | No |
| 267 | CREBZF |  |  | 1464 | 0.490 | 0.4967 | No |
| 268 | ARHGAP11B |  |  | 1471 | 0.488 | 0.4969 | No |
| 269 | EXOSC9 |  |  | 1472 | 0.487 | 0.4983 | No |
| 270 | CKS1B |  |  | 1502 | 0.479 | 0.4937 | No |
| 271 | CEP55 |  |  | 1508 | 0.479 | 0.4940 | No |
| 272 | C5orf34 |  |  | 1514 | 0.478 | 0.4944 | No |
| 273 | C2orf69 |  |  | 1516 | 0.478 | 0.4955 | No |
| 274 | EMC9 |  |  | 1522 | 0.477 | 0.4959 | No |
| 275 | LSM8 |  |  | 1524 | 0.476 | 0.4970 | No |
| 276 | HIST1H1A |  |  | 1533 | 0.473 | 0.4968 | No |
| 277 | KAT2B |  |  | 1537 | 0.472 | 0.4975 | No |
| 278 | KMT2A |  |  | 1540 | 0.471 | 0.4984 | No |
| 279 | IQGAP3 |  |  | 1546 | 0.471 | 0.4988 | No |
| 280 | SRRT |  |  | 1563 | 0.467 | 0.4968 | No |
| 281 | KIAA0586 |  |  | 1566 | 0.466 | 0.4977 | No |
| 282 | RNFT2 |  |  | 1573 | 0.464 | 0.4978 | No |
| 283 | DLEU2 |  |  | 1587 | 0.462 | 0.4965 | No |
| 284 | G2E3 |  |  | 1602 | 0.459 | 0.4949 | No |
| 285 | ZEB1 |  |  | 1605 | 0.459 | 0.4958 | No |
| 286 | PHF6 |  |  | 1633 | 0.452 | 0.4915 | No |
| 287 | BORA |  |  | 1648 | 0.447 | 0.4899 | No |
| 288 | IFT122 |  |  | 1650 | 0.447 | 0.4910 | No |
| 289 | TUBB4B |  |  | 1659 | 0.444 | 0.4906 | No |
| 290 | BRD8 |  |  | 1694 | 0.437 | 0.4848 | No |
| 291 | CALM3 |  |  | 1710 | 0.435 | 0.4830 | No |
| 292 | HYLS1 |  |  | 1711 | 0.434 | 0.4842 | No |
| 293 | TBL1XR1 |  |  | 1712 | 0.434 | 0.4855 | No |
| 294 | ZNF639 |  |  | 1718 | 0.434 | 0.4857 | No |
| 295 | RNF4 |  |  | 1726 | 0.432 | 0.4855 | No |
| 296 | ESPL1 |  |  | 1733 | 0.430 | 0.4855 | No |
| 297 | TDP1 |  |  | 1748 | 0.428 | 0.4839 | No |
| 298 | GLCCI1 |  |  | 1764 | 0.425 | 0.4820 | No |
| 299 | UCP2 |  |  | 1779 | 0.422 | 0.4803 | No |
| 300 | WWTR1 |  |  | 1794 | 0.419 | 0.4786 | No |
| 301 | NET1 |  |  | 1801 | 0.417 | 0.4786 | No |
| 302 | VEZF1 |  |  | 1804 | 0.417 | 0.4794 | No |
| 303 | ELF2 |  |  | 1806 | 0.416 | 0.4803 | No |
| 304 | KBTBD2 |  |  | 1810 | 0.416 | 0.4809 | No |
| 305 | QSER1 |  |  | 1813 | 0.416 | 0.4817 | No |
| 306 | METTL4 |  |  | 1820 | 0.415 | 0.4817 | No |
| 307 | SNRPG |  |  | 1825 | 0.414 | 0.4820 | No |
| 308 | CRNDE |  |  | 1860 | 0.408 | 0.4762 | No |
| 309 | CEP44 |  |  | 1918 | 0.401 | 0.4655 | No |
| 310 | CARD8 |  |  | 1952 | 0.396 | 0.4598 | No |
| 311 | CENPW |  |  | 1959 | 0.395 | 0.4597 | No |
| 312 | LSM5 |  |  | 1982 | 0.391 | 0.4563 | No |
| 313 | TMX1 |  |  | 2012 | 0.386 | 0.4514 | No |
| 314 | PPM1G |  |  | 2017 | 0.385 | 0.4517 | No |
| 315 | EPHA4 |  |  | 2034 | 0.382 | 0.4495 | No |
| 316 | CEP70 |  |  | 2041 | 0.382 | 0.4494 | No |
| 317 | MSI2 |  |  | 2042 | 0.382 | 0.4505 | No |
| 318 | DAXX |  |  | 2044 | 0.381 | 0.4514 | No |
| 319 | GTPBP1 |  |  | 2071 | 0.377 | 0.4471 | No |
| 320 | CEP57L1 |  |  | 2082 | 0.375 | 0.4461 | No |
| 321 | XPO1 |  |  | 2091 | 0.374 | 0.4455 | No |
| 322 | ANLN |  |  | 2125 | 0.371 | 0.4397 | No |
| 323 | NUP35 |  |  | 2127 | 0.370 | 0.4406 | No |
| 324 | MDC1 |  |  | 2139 | 0.369 | 0.4394 | No |
| 325 | NUP205 |  |  | 2169 | 0.366 | 0.4344 | No |
| 326 | ABAT |  |  | 2170 | 0.365 | 0.4355 | No |
| 327 | C4orf46 |  |  | 2172 | 0.365 | 0.4363 | No |
| 328 | NGLY1 |  |  | 2174 | 0.365 | 0.4372 | No |
| 329 | HNRNPR |  |  | 2175 | 0.365 | 0.4382 | No |
| 330 | PNRC2 |  |  | 2176 | 0.365 | 0.4393 | No |
| 331 | C12orf57 |  |  | 2177 | 0.364 | 0.4403 | No |
| 332 | RHEB |  |  | 2192 | 0.363 | 0.4385 | No |
| 333 | LDHB |  |  | 2219 | 0.360 | 0.4341 | No |
| 334 | HSPA13 |  |  | 2238 | 0.358 | 0.4314 | No |
| 335 | BSG |  |  | 2255 | 0.356 | 0.4292 | No |
| 336 | CSTF1 |  |  | 2274 | 0.353 | 0.4265 | No |
| 337 | CDK19 |  |  | 2291 | 0.351 | 0.4242 | No |
| 338 | NUDCD2 |  |  | 2311 | 0.348 | 0.4212 | No |
| 339 | ASXL1 |  |  | 2339 | 0.343 | 0.4166 | No |
| 340 | WDPCP |  |  | 2348 | 0.342 | 0.4160 | No |
| 341 | SKIDA1 |  |  | 2350 | 0.342 | 0.4167 | No |
| 342 | TGIF2 |  |  | 2359 | 0.341 | 0.4161 | No |
| 343 | ANAPC11 |  |  | 2420 | 0.332 | 0.4046 | No |
| 344 | CHCHD3 |  |  | 2434 | 0.330 | 0.4029 | No |
| 345 | TOB1 |  |  | 2442 | 0.330 | 0.4024 | No |
| 346 | SKA2 |  |  | 2445 | 0.329 | 0.4029 | No |
| 347 | ARHGAP19 |  |  | 2451 | 0.328 | 0.4028 | No |
| 348 | CCNB1 |  |  | 2480 | 0.325 | 0.3980 | No |
| 349 | HNRNPA2B1 |  |  | 2523 | 0.320 | 0.3902 | No |
| 350 | FOPNL |  |  | 2526 | 0.320 | 0.3907 | No |
| 351 | TMEM60 |  |  | 2576 | 0.314 | 0.3815 | No |
| 352 | COX6C |  |  | 2583 | 0.313 | 0.3811 | No |
| 353 | CEP85 |  |  | 2585 | 0.313 | 0.3818 | No |
| 354 | SFRP1 |  |  | 2591 | 0.312 | 0.3817 | No |
| 355 | AHCY |  |  | 2598 | 0.311 | 0.3813 | No |
| 356 | HMGN3 |  |  | 2616 | 0.308 | 0.3787 | No |
| 357 | TOPORS |  |  | 2641 | 0.305 | 0.3746 | No |
| 358 | DCXR |  |  | 2647 | 0.305 | 0.3745 | No |
| 359 | SF3B3 |  |  | 2649 | 0.305 | 0.3751 | No |
| 360 | RNF26 |  |  | 2660 | 0.303 | 0.3739 | No |
| 361 | PPP2R5C |  |  | 2702 | 0.299 | 0.3663 | No |
| 362 | SRSF3 |  |  | 2732 | 0.297 | 0.3612 | No |
| 363 | RUVBL1 |  |  | 2739 | 0.296 | 0.3608 | No |
| 364 | PET100 |  |  | 2770 | 0.292 | 0.3554 | No |
| 365 | TGS1 |  |  | 2781 | 0.291 | 0.3542 | No |
| 366 | NDC1 |  |  | 2782 | 0.291 | 0.3550 | No |
| 367 | CDK16 |  |  | 2797 | 0.289 | 0.3530 | No |
| 368 | VDAC3 |  |  | 2803 | 0.289 | 0.3528 | No |
| 369 | BUB3 |  |  | 2806 | 0.288 | 0.3532 | No |
| 370 | GCA |  |  | 2870 | 0.283 | 0.3409 | No |
| 371 | SAE1 |  |  | 2872 | 0.283 | 0.3416 | No |
| 372 | PPP1CC |  |  | 2903 | 0.278 | 0.3361 | No |
| 373 | TTLL5 |  |  | 2908 | 0.278 | 0.3361 | No |
| 374 | ODF2 |  |  | 2911 | 0.278 | 0.3365 | No |
| 375 | CNTROB |  |  | 2918 | 0.277 | 0.3361 | No |
| 376 | CPSF3 |  |  | 2932 | 0.276 | 0.3342 | No |
| 377 | NIF3L1 |  |  | 2969 | 0.272 | 0.3275 | No |
| 378 | ABHD10 |  |  | 2989 | 0.271 | 0.3244 | No |
| 379 | COX8A |  |  | 2998 | 0.270 | 0.3235 | No |
| 380 | KATNBL1 |  |  | 3000 | 0.269 | 0.3240 | No |
| 381 | RANGAP1 |  |  | 3006 | 0.268 | 0.3238 | No |
| 382 | PTPRE |  |  | 3014 | 0.267 | 0.3231 | No |
| 383 | HDAC6 |  |  | 3048 | 0.264 | 0.3170 | No |
| 384 | TNFAIP8L1 |  |  | 3081 | 0.261 | 0.3112 | No |
| 385 | NRF1 |  |  | 3093 | 0.261 | 0.3096 | No |
| 386 | RCCD1 |  |  | 3103 | 0.260 | 0.3085 | No |
| 387 | FAM72D |  |  | 3107 | 0.260 | 0.3087 | No |
| 388 | VRK3 |  |  | 3132 | 0.257 | 0.3044 | No |
| 389 | HSPA2 |  |  | 3149 | 0.255 | 0.3018 | No |
| 390 | UXT |  |  | 3199 | -0.251 | 0.2924 | No |
| 391 | FAM160B1 |  |  | 3200 | -0.251 | 0.2932 | No |
| 392 | PSME1 |  |  | 3202 | -0.251 | 0.2937 | No |
| 393 | OXA1L |  |  | 3235 | -0.257 | 0.2878 | No |
| 394 | ITGB1BP1 |  |  | 3341 | -0.274 | 0.2668 | No |
| 395 | GOT1 |  |  | 3406 | -0.283 | 0.2544 | No |
| 396 | PSMB3 |  |  | 3413 | -0.284 | 0.2540 | No |
| 397 | SAV1 |  |  | 3464 | -0.293 | 0.2445 | No |
| 398 | TRMT112 |  |  | 3474 | -0.295 | 0.2435 | No |
| 399 | HADHA |  |  | 3497 | -0.300 | 0.2398 | No |
| 400 | DYNC2H1 |  |  | 3530 | -0.305 | 0.2340 | No |
| 401 | RBM8A |  |  | 3571 | -0.311 | 0.2266 | No |
| 402 | RNF181 |  |  | 3684 | -0.328 | 0.2044 | No |
| 403 | RPL7 |  |  | 3704 | -0.332 | 0.2014 | No |
| 404 | RPL8 |  |  | 3733 | -0.336 | 0.1966 | No |
| 405 | DR1 |  |  | 3888 | -0.365 | 0.1658 | No |
| 406 | PABPC1 |  |  | 3893 | -0.366 | 0.1660 | No |
| 407 | TUBB6 |  |  | 3904 | -0.368 | 0.1650 | No |
| 408 | RPS27L |  |  | 3927 | -0.373 | 0.1615 | No |
| 409 | CCT7 |  |  | 3954 | -0.377 | 0.1572 | No |
| 410 | TUBA1C |  |  | 3957 | -0.379 | 0.1579 | No |
| 411 | CCT5 |  |  | 3958 | -0.379 | 0.1590 | No |
| 412 | COPZ1 |  |  | 3990 | -0.387 | 0.1537 | No |
| 413 | DYRK4 |  |  | 4123 | -0.415 | 0.1276 | No |
| 414 | LDHA |  |  | 4145 | -0.421 | 0.1244 | No |
| 415 | ZNF618 |  |  | 4186 | -0.434 | 0.1174 | No |
| 416 | RPL36AL |  |  | 4201 | -0.440 | 0.1158 | No |
| 417 | HMGCR |  |  | 4221 | -0.446 | 0.1131 | No |
| 418 | RPS3A |  |  | 4266 | -0.462 | 0.1053 | No |
| 419 | RPL23A |  |  | 4295 | -0.471 | 0.1009 | No |
| 420 | F2R |  |  | 4316 | -0.476 | 0.0981 | No |
| 421 | TOP1 |  |  | 4335 | -0.483 | 0.0958 | No |
| 422 | CD99 |  |  | 4339 | -0.484 | 0.0966 | No |
| 423 | B2M |  |  | 4341 | -0.485 | 0.0978 | No |
| 424 | EIF3F |  |  | 4349 | -0.486 | 0.0977 | No |
| 425 | MORF4L2 |  |  | 4354 | -0.488 | 0.0983 | No |
| 426 | PSME2 |  |  | 4422 | -0.508 | 0.0859 | No |
| 427 | GDI2 |  |  | 4448 | -0.519 | 0.0822 | No |
| 428 | PDCD4 |  |  | 4460 | -0.524 | 0.0815 | No |
| 429 | COX7A2L |  |  | 4473 | -0.528 | 0.0805 | No |
| 430 | RPLP0 |  |  | 4483 | -0.532 | 0.0802 | No |
| 431 | TJP1 |  |  | 4489 | -0.536 | 0.0807 | No |
| 432 | PRR13 |  |  | 4519 | -0.548 | 0.0763 | No |
| 433 | MBNL2 |  |  | 4558 | -0.569 | 0.0700 | No |
| 434 | ARL4A |  |  | 4593 | -0.582 | 0.0647 | No |
| 435 | PSMB2 |  |  | 4594 | -0.583 | 0.0664 | No |
| 436 | ANXA5 |  |  | 4616 | -0.601 | 0.0638 | No |
| 437 | RPS3 |  |  | 4632 | -0.610 | 0.0624 | No |
| 438 | RPL6 |  |  | 4649 | -0.618 | 0.0609 | No |
| 439 | LLPH |  |  | 4652 | -0.619 | 0.0623 | No |
| 440 | RPL12 |  |  | 4657 | -0.621 | 0.0632 | No |
| 441 | EIF3D |  |  | 4676 | -0.631 | 0.0613 | No |
| 442 | AAMDC |  |  | 4691 | -0.641 | 0.0603 | No |
| 443 | MSN |  |  | 4698 | -0.644 | 0.0609 | No |
| 444 | HSPA5 |  |  | 4714 | -0.652 | 0.0597 | No |
| 445 | EIF5A |  |  | 4746 | -0.676 | 0.0552 | No |
| 446 | RPS6 |  |  | 4751 | -0.678 | 0.0563 | No |
| 447 | ACTN1 |  |  | 4775 | -0.695 | 0.0536 | No |
| 448 | PIM1 |  |  | 4783 | -0.699 | 0.0541 | No |
| 449 | LGALS1 |  |  | 4784 | -0.699 | 0.0562 | No |
| 450 | RAB13 |  |  | 4786 | -0.701 | 0.0580 | No |
| 451 | EIF3E |  |  | 4804 | -0.718 | 0.0565 | No |
| 452 | NACA |  |  | 4809 | -0.721 | 0.0578 | No |
| 453 | STK17B |  |  | 4824 | -0.734 | 0.0570 | No |
| 454 | PKM |  |  | 4828 | -0.737 | 0.0585 | No |
| 455 | SLC3A2 |  |  | 4861 | -0.765 | 0.0541 | No |
| 456 | SNAPC1 |  |  | 4862 | -0.768 | 0.0563 | No |
| 457 | CLIC1 |  |  | 4899 | -0.809 | 0.0512 | No |
| 458 | RPS27A |  |  | 4909 | -0.822 | 0.0517 | No |
| 459 | LMO7 |  |  | 4925 | -0.836 | 0.0510 | No |
| 460 | ZFP36L1 |  |  | 4929 | -0.843 | 0.0528 | No |
| 461 | ACTG1 |  |  | 5017 | -0.961 | 0.0376 | No |
| 462 | NPM1 |  |  | 5040 | -1.017 | 0.0360 | No |
| 463 | DDX10 |  |  | 5061 | -1.053 | 0.0349 | No |
| 464 | ECI2 |  |  | 5064 | -1.065 | 0.0375 | No |
| 465 | ADAMTS6 |  |  | 5117 | -1.196 | 0.0302 | No |
| 466 | FBN1 |  |  | 5263 | -2.234 | 0.0066 | No |
Table: GSEA details [plain text format]

  

Fig 2: RICHARDS\_NOWAKOWSKI\_SCIENCE\_2017\_MGE\_IPC1\_UPREG: Random ES distribution      
 Gene set null distribution of ES for **RICHARDS\_NOWAKOWSKI\_SCIENCE\_2017\_MGE\_IPC1\_UPREG**
